# Insights on Structural Characteristics and Ligand Binding Mechanisms of CDK2

**DOI:** 10.3390/ijms16059314

**Published:** 2015-04-24

**Authors:** Yan Li, Jingxiao Zhang, Weimin Gao, Lilei Zhang, Yanqiu Pan, Shuwei Zhang, Yonghua Wang

**Affiliations:** 1 Key laboratory of Industrial Ecology and Environmental Engineering (MOE), Faculty of Chemical, Environmental and Biological Science and Technology, Dalian University of Technology, Dalian 116024, China; E-Mails: d11107022@mail.dlut.edu.cn (J.Z.); weimingao@mail.dlut.edu.cn (W.G.); yqpan@dlut.edu.cn (Y.P.); zswei@dlut.edu.cn (S.Z.); 2 School of Chemistry and Environmental Engineering, Hubei University for Nationalities, Enshi 445000, China; E-Mail: zhanglilei@outlook.com; 3 Center of Bioinformatics, College of Life Science, Northwest A&F University, Yangling 712100, China

**Keywords:** CDK2 (cyclin-dependent kinase 2), binding mechanism, variations

## Abstract

Cyclin-dependent kinase 2 (CDK2) is a crucial regulator of the eukaryotic cell cycle. However it is well established that monomeric CDK2 lacks regulatory activity, which needs to be aroused by its positive regulators, cyclins E and A, or be phosphorylated on the catalytic segment. Interestingly, these activation steps bring some dynamic changes on the 3D-structure of the kinase, especially the activation segment. Until now, in the monomeric CDK2 structure, three binding sites have been reported, including the adenosine triphosphate (ATP) binding site (Site I) and two non-competitive binding sites (Site II and III). In addition, when the kinase is subjected to the cyclin binding process, the resulting structural changes give rise to a variation of the ATP binding site, thus generating an allosteric binding site (Site IV). All the four sites are demonstrated as being targeted by corresponding inhibitors, as is illustrated by the allosteric binding one which is targeted by inhibitor ANS (fluorophore 8-anilino-1-naphthalene sulfonate). In the present work, the binding mechanisms and their fluctuations during the activation process attract our attention. Therefore, we carry out corresponding studies on the structural characterization of CDK2, which are expected to facilitate the understanding of the molecular mechanisms of kinase proteins. Besides, the binding mechanisms of CDK2 with its relevant inhibitors, as well as the changes of binding mechanisms following conformational variations of CDK2, are summarized and compared. The summary of the conformational characteristics and ligand binding mechanisms of CDK2 in the present work will improve our understanding of the molecular mechanisms regulating the bioactivities of CDK2.

## 1. Introduction

Cyclin-dependent kinases (CDKs), which belong to an important affiliated subfamily of Ser/Thr protein kinases, have been extensively studied due to their essential roles in cell division cycle, transcription, differentiation, neuronal functions, as well as apoptosis [1]. To date, 13 CDK family members (CDK1–CDK13) have been identified [2]. While several CDKs like CDK2 are well studied and plenty of structural and biochemical information are available, others such as CDK12 and CDK13, have only recently been characterized [2,3]. All CDKs are similar in size (30–40 kDa) and share at least 40% homology [4].

It is well established that the CDK monomers are inactive in the quiescent cells, and are activated successively by specific cyclin partners at the proper point of time in the cell cycle [5]. The binding of cyclin subunits confers basal kinase activity to the complex [6]. For example, cyclin A binding to CDK2 relieves the blocking of the catalytic cleft, and also exposes the phosphorylation site (Thr160) which sets the stage for the full activation of CDK2. Besides CDK2, several other CDKs, including CDK1, 4, 5 and 6, also need to be phosphorylated on a conserved threonine residue within the activation segment (also known as the T-loop) [7,8,9,10]. These activated processes can result in conformational changes in and around the catalytic domain of CDKs, which indicates the intrinsic conformational flexibility of CDKs [11]. This flexibility plays a central role in permitting CDKs to be regulated in several different ways, as well as in switching the states of CDKs in response to various regulatory signals which are involved in the growth of the eukaryotic cell [11].

A “superstar” among the CDK family is CDK2. As a matter of fact, the up-to-date number of CDK2-related crystal structures (including both CDK2 and CDK2/cyclin complexes with or without substrates) recorded in the Protein Data Bank database (PDB, http://www.rcsb.org/pdb/home/home.do) is over 300 [12], far exceeding the total number of other CDK members uploaded to this database. It is established that CDK2 plays a pivotal role in the promotion of the cell cycle progression. In synergy with its positive regulatory subunits of cyclins E and A, respectively, this evolutionarily conserved kinase promotes the transition of the well-established G_1_/S phase boundary and drives the cell cycle through S interval [13,14,15]. In detail, CDK2/cyclin A is required for orderly S-phase progression [14], while CDK2/cyclin E complex mediates the phosphorylation of retinoblastoma protein to facilitate the G1/S transition [15]. The distinguishing role of CDK2 may be its close association with cancers, and it is also reported that a number of other diseases in correlation with cell disorder can arise from, or involve improper regulation of, these kinase activities [16]. Aberrant CDK2 activity (hyperactivation of CDK, underexpression and aberrant expression of cyclins) may lead to the loss of cell control, hence inducing infinite proliferation of cell [17], which is a common feature of most malignancies. Factually, CDK2 has been found to play a pivotal role in cell proliferation of prostate cancer [18] and non-small cell cancer [19], and is also crucial for malignant transformation of breast epithelial cells. Suppression of CDK2 activity can effectively inhibit the proliferation of human breast cancer cells [20], which also includes those resistant to endocrinotherapy [21]. Moreover, unchecked CDK2 activity which is persistent E2F transcriptional activity unless terminated during S phase, constitutes a potent apoptotic signal [22]. Thus, the reinstatement of CDK inhibition gives a chance to pharmacological interference with tumor progression. Therefore, given its critical role in cell cycle regulation, CDK2 has been actively considered as a potential drug target for anticancer therapeutics [13].

**Figure 1:**
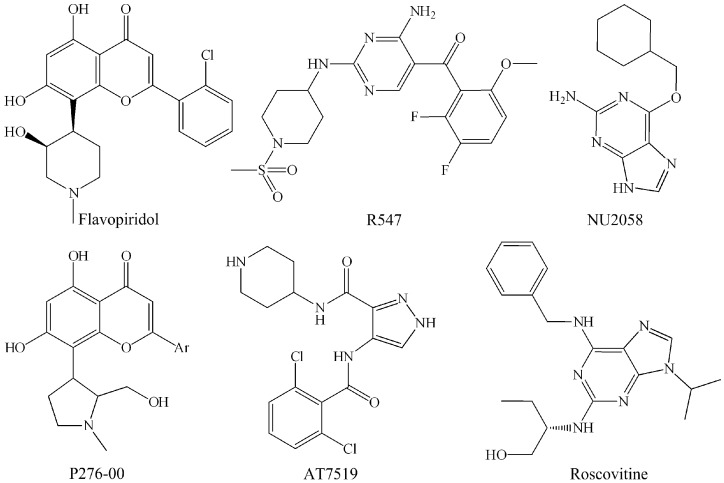
ATP-competitive CDK2 inhibitors.

Anticancer therapies a few decades ago began with the crystal structures of the single protein and their complexes. Currently, however, designing biologically active small molecules like CDK inhibitors (CDKIs) is becoming a more and more legitimate solution to cell proliferation. Thus far, plenty of CDK2 inhibitors have been discovered, and they can be subdivided into ATP-competitive and non-ATP-competitive inhibitors according to their specific target binding sites [23]. Among them, the ATP-competitive inhibitors are most extensively investigated, and some of them are evaluated by various stages of clinical trials [24] which are displayed in Table 1. For example, the first generation of ATP-competitive inhibitors includes flavopiridol, R547 [25] and roscovitine [26] (as displayed in Figure 1), which are already undergoing first or second-stage clinical trials. Nevertheless, it is reported that this class of inhibitors does not match clinical trial expectations because of its low activities and/or toxicities [23]. The major reason is that the low specificity exhibited by these inhibitors often results in off-target effects, thus ultimately limiting their therapeutic efficiencies [27]. Therefore, the second generation of ATP-competitive inhibitors are more potent, and specific ligands for CDKs such as P276-00 [28], AT7519 [29] and NU2058 [30] (Figure 1), have been developed and assessed in preclinical and clinical trials. For instance, a highly specific inhibitor of CDK2 (IC_50_ = 10 nM) [31], P276-00, exhibits potent anti-proliferative effects against several human tumor cells [32], and is undergoing phase I–II clinical trials [33]. However, the high sequence homology within the ATP binding sites of cellular kinases makes the work of designing small molecules like P276-00, which can effectively target CDK2 at its ATP binding site, more complex [27]. Therefore, besides the CDK2/cyclin A, P276-00 also has nanomolar activities in CDK4/cyclin D1, CDK1/cyclin B and CDK9/cyclin T1 [27]. However, it is noteworthy that non-ATP competitive inhibitors, the other type of CDK2 inhibitors, which inhibit the substrates of CDK2−cyclin complexes and regulatory binding sites, have high specificity [23]. Currently, several non-ATP competitive CDK2 inhibitors which show great potential for clinical applications have been developed, including Spa310 (a 39-residue peptide) and CIP (a p53-derived peptide) [34]. Taking Spa310 as an example, this molecule can inhibit CDK2 activity and significantly reduce the cellular proliferation of NIH3T3 [35]. Therefore, analyzing the binding modes of these ligands as well as the structural characteristics of CDK2 can enhance our understanding of the molecular mechanisms regulating the activities of this protein kinase [36].

**Table 1:** CDK2 inhibitors in clinical trials.

Candidate Drug	Target (IC_50_)	Clinical Trials	References
Flavopiridol	CDK1 (30 nM), CDK2 (100 nM), CDK4 (20 nM), CDK6 (60 nM), CDK7 (10 nM), CDK9 (10 nM)	Phase II	Kaur *et al.* [37]
P276-00	CDK1 (110 nM), CDK2 (10 nM), CDK4 (130 nM), CDK9 (20 nM)	Phase II	De Azevedo Jr. *et al.* [38]; Murthi *et al.* [39]; Joshi *et al.* [33]
Roscovitine	CDK1 (2.7 μM), CDK2 (0.7 μM), CDK7 (0.5 μM), CDK9 (0.8 μM)	Phase II	McClue *et al.* [40]; Meijer *et al.* [41]
PHA-848125 AC	CDK1 (2 nM), CDK2 (3 nM), CDK4 (5 nM), CDK5 (4 nM)	Phase II	Caporali *et al.* [42]
UCN-01	CDK2 (42 nM), CDK4 (32 nM), CDK6 (58 nM)	Phase II	Kawakami *et al.* [43]; Li *et al.* [44]; Senderowicz [45]
R547	CDK1 (0.001 μM), CDK2 (0.003 μM), CDK4 (0.001 μM)	Phase I/II	Chu *et al.* [46]; DePinto *et al.* [25]
AT-7519	CDK1 (0.21 μM), CDK2 (0.047 μM), CDK4 (0.1 μM), CDK5 (0.13 μM), CDK6 (0.17 μM), CDK9 (≤0.01 μM)	Phase I/II	Mahadevan *et al.* [47]; Squires *et al.* [48]
Dinaciclib	CDK1 (3 nM), CDK2 (1 nM), CDK5 (1 nM), CDK9 (4 nM)	Phase I	Michael [49]; Parry *et al.* [50]
SNS-032	CDK2 (38 nM), CDK7 (62 nM), CDK9 (4 nM)	Phase I	Tong *et al.* [51]; Conroy *et al.* [52]
RGB-286638	CDK1 (2 nM), CDK2 (3 nM), CDK3 (5 nM), CDK4 (4 nM), CDK9 (1 nM)	Phase I	de Bruijn *et al.* [53]; van der Biessen *et al.* [54]
BAY-1000394	CDK1, CDK2, CDK4 and CDK9 (≤11 nM)	Phase I	Siemeister *et al.* [55]; Siemeister *et al.* [56]
TG02	CDK1 (9 nM), CDK2 (5 nM), CDK3 (8 nM), CDK5 (4 nM), CDK9 (3 nM)	Phase I	Poulsen *et al.* [57]

Noticeably, CDK2 will undergo some structural changes during the activation process by cyclin binding and phosphorylation on the activation segment, which results in the variation of the ATP binding site and simultaneously generates a new allosteric binding site. The conformational variations of CDK2 also cause the structural changes of these sites, and the change mechanisms as well as the binding mode of these sites attract our attention. Therefore, in this review, we will provide an overview of CDK2 and relevant inhibitors with a focus on the fluctuations of the structure of this kinase, and then discuss the binding mechanisms of inhibitors with CDK2.

## 2. Binding Sites of the Monomeric Cyclin-Dependent Kinase 2 (CDK2) and the CDK2/Cyclin Complexes

CDK2 promotes the G_1_/S boundary checkpoint and drives the cell cycle through the S phase by the bindings of cyclins E and A, respectively [58,59]. The overexpression of CDK2 may lead to loss of cell control. However, if there is no corresponding cyclin, CDK2 will not be transiently activated to take effects [5]. The incorporation of the cyclin subunit on one side of the catalytic cleft connecting both the *N*- and *C*-terminal lobes of CDK2, and the phosphorylation on Thr160 forms a large, continuous protein−protein interface [60,61]. Interestingly, neither cyclin binding nor phosphorylation alone is sufficient to achieve full activation of CDK2 [60,62,63,64,65]. Considering this fact, inhibition or disruption of the CDK2/cyclin complexes should be feasible to suppress the hyperactivation of CDK2 and hold back the infinite cell proliferation.

### 2.1. Characterization of Monomeric CDK2

**Figure 2:**
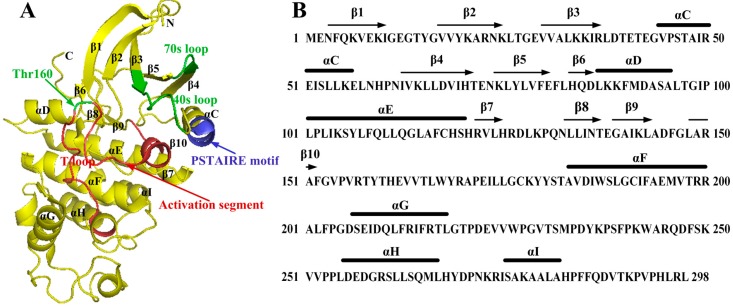
The structure of monomeric CDK2. (**A**) Schematic diagram showing the structure of monomeric CDK2 using coordinates 4KD1. Secondary structural elements and the important fragments are labeled; (**B**) Sequences of CDK2 showing secondary structural elements.

As a typical protein kinase, the monomeric CDK2 consists of 298 amino acids (PDB code: 1HCL, residues 1–298), and folds into a typical bilobal structure, with a smaller *N*-terminal lobe (residues 1–85) that contains an antiparallel five-strand β-sheet and a major C-helix, together with a larger *C*-terminal lobe predominantly composed of α-helix [66]. Actually, the two terminal domains are connected through a single peptide strand (residues 81–83) which acts as a hinge linker to ensure that the two lobes can rotate with respect to each other without disruption of the secondary structure of this kinase [67]. Figure 2 depicts the structure of monomeric CDK2. As displayed in this figure, there are two important segments in the large *C*-terminal domain, including the catalytic residues that are in charge of phosphorylation promotion, as well as the activation segment. Among them, this activation segment spans residues between the conserved DFG (residues 145–147) and APE motifs (residues 170–172), and also includes the phosphorylation site, *i.e.*, residue Thr160 [68]. In the inert CDK2 monomer (PDB code: 1HCK), the Thr160 among the activation segment conformation is buried away from solvent facing toward the conserved glycine-rich loop [69]. The studies on structure [6] point out that the activation loop of monomeric unphosphorylated CDK2 (PDB code: 1WCC) can adopt a range of conformations which are distinct from the inert CDK2 monomer [70]. The T-loop which is located at the entrance of the catalytic cleft, holds back the polypeptide substrates and, in this way, prevents them from entering into the cleft to interact with ATP [71]. In detail, the side chain of the catalytic site residue Glu51, which is ensconced in the PSTAIRE helix, lies toward outside the catalysis cleft [72]. Additionally, the arginine residues (Arg50, Arg126 and Arg150) in the catalytic cleft, especially for those in the putative polypeptide substrate active site, are also of great importance for the binding ability with the phosphate group of ATP [11,73]. The unique sequence of the CDK family is the PSTAIRE motif (residues 45–51) embodied in the *N*-terminal C-helix, which has a key role in the interface. Thus far, four binding sites in the CDK2 structure and one binding cavity in cyclin A have been discovered. Among the four sites on CDK2, one shows up only after particular structural transformation, while the other three can be found on the original CDK2.

**I. Competitive binding site (Site I).** The most well-known ATP binding site, located deep at the junction of the N and C domains, includes the pivotal catalytic residues with high sensitivity and linker region (residues 81–83), and consists of 136 consecutive amino acids (10–145) on the CDK2 [74]. This pocket is highly characterized by a hydrophobic feature and tends to bind a highly polar/charged heterocyclic molecule [75,76,77]. Various inhibitors bind to the ATP binding pocket and normally occupy the adenine ring (subsite) of the ATP. In fact, it is well established that the ATP binding site is conservative [78], and ATP recognition involves residues from both N and C lobes. Small agents inhibit CDK2 through competition with ATP for the catalytic site to hit the mark of catalytic subunit. Researchers sort this site to six subsections: (1) the adenine region; (2) the ribose pocket; (3) the triphosphate binding region; (4) hydrophobic region I (opposite of the ribose pocket); (5) hydrophobic region II (cleft adjacent to the ribose pocket) and (6) the hinge region (connecting the *N*- and *C*-terminal domains) [16], or simply the adenine, ribose and triphosphate subsites. The hydrogen bonds formed with the hinge region of the kinase are set as the primary criteria to appraise a multitude of inhibitors as all known inhibitors form hydrogen bonds with the linker region of CDKs’ ATP binding pocket [79]. Nevertheless, it is worth noting that, owing to high conservation of the PSTAIRE helix among CDKs, the ATP site is not the perfect binding pocket for highly specific inhibitors. In fact, this group of CDK2 inhibitors is always accompanied by off-target effects, ultimately limiting their therapeutic efficiencies.

**Figure 3:**
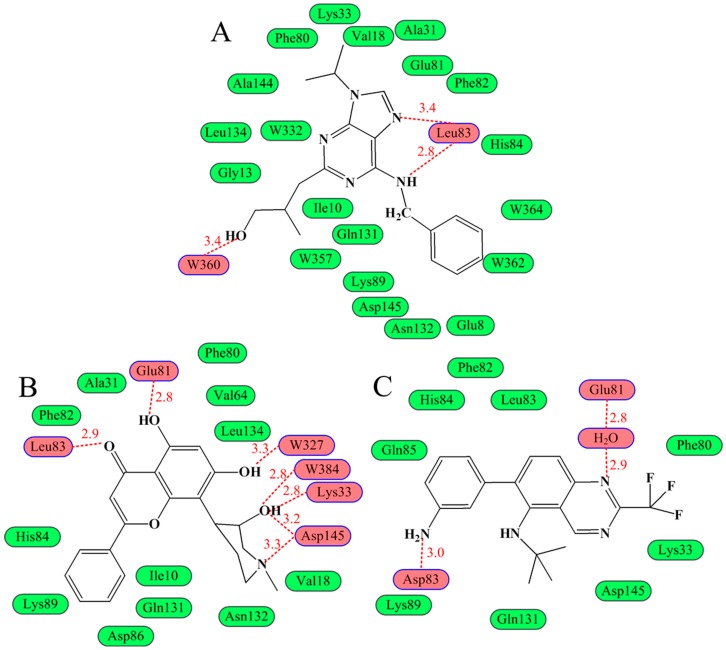
Three binding modes in ATP pocket of CDK2 [80]. The ligands in (**A**); (**B**,**C**) are roscovitine, flavopiridol and a 4-anilinoquinazoline derivative, respectively. Green nodes represent the residues around the ligands, while pink ones signify those residues and water molecules involved in the H-bond network. Hydrogen bonds are indicated by broken lines with the distance in Å.

**Figure 4:**
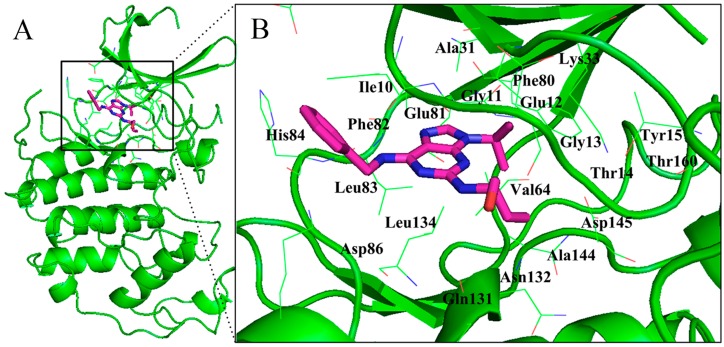
Position of competitive binding site. Panel (**A**) displays the competitive binding site of the monomer CDK2 with inhibitor roscovitine (coordinates 2A4L); the detailed information of amino acids in this cavity is shown in panel (**B**).

Three binding modes in the ATP binding site of CDK2 have already been summarized in our previous work [80]. The purine derivative roscovitine represents the first binding mode as displayed in Figure 3A [34]. The core portion of these inhibitors roughly overlaps with the adenine region, and these ligands’ binding does not bring substantial change to the domain orientations when compared with the ATP–enzyme complex. However, the two ring systems have a rotation with respect to ATP, and this movement allows inhibitors to have contacts with the enzyme, which is not observed in the ATP bond. Take roscovitine as an example, where the benzyl ring occupies a region that does not hold onto any part of the ATP as shown in Figure 4. The three conservative H-bonds formed with the backbones of residues Glu81 and Leu83 are cardinals, at least two of which can be observed in this binding fashion. In addition to H-bonds, hydrophobic and van der Waals interactions are also important complements in this mechanism. Most of the scaffolds following this binding mode are similar to the purine skeleton. The key representative flavonoid molecule flavopiridol stands for the second binding mode (Figure 3B) [1]. The benzopyran ring of the flavopiridol adopts a discrepant orientation with both ATP and the purine analogs, and the atomic interaction is also quite different. Two of the conserved H-bonds can also be observed in this H-bond network; however, residues Lys33 and Asp145 that are respectively located in the ribose and triphosphate regions of ATP also come in to connect with the ligand. What is more, two water molecules within the receptor play important roles in the interaction. Furthermore, the 4-anilinoquinazoline class of inhibitors displays the third interaction mechanism as displayed in Figure 3C. Accommodation of this series of inhibitors also does not change the conformation of CDK2 greatly, with only some side chain alterations taking place in several residues (such as Lys33 and Lys89), and the backbones of the residues with respect to the enzyme have not changed [81]. Within a scope of 3.5 Å, the key binding elements are Lys33, Phe80, Leu83, His84, Asp86, Lys89 and Asp145. The acting forces to fix the ligand are a water-bridged H-bond with the backbone oxygen of residue Glu81, an additional hydrogen bond with the side chain oxygen of Asp86, as well as the stacking interaction with the gatekeeper Phe80 [82].

**Figure 5:**
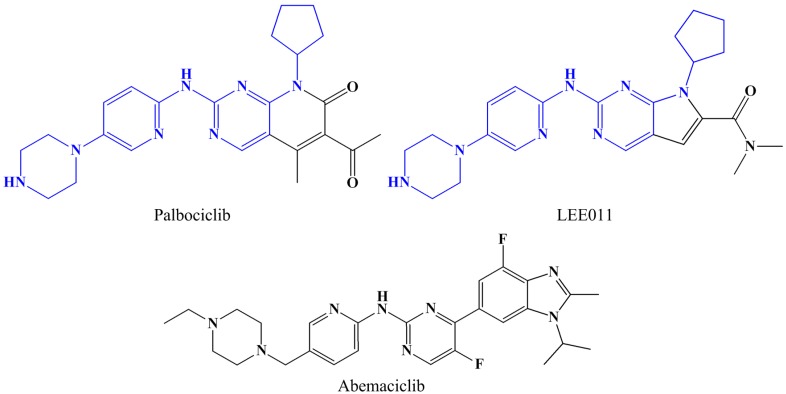
Three CDK4/CDK6 inhibitors in clinics.

All potent CDK inhibitors act by competing with ATP or blocking ATP binding for having interaction with the catalytic site of CDK [74]. However, due to the fact that the ATP binding site of the human kinases is well conserved [83], the high selective inhibitors of CDK2 are, though in dire need, quite difficult to obtain. As for CDK4/6 kinase, some of its selective inhibitors, like palbociclib (PD0332991; Pfizer), LEE011 (Novartis) and abemaciclib (LY2835219; Eli Lilly), are already under the examination process in clinical trials, as displayed in Figure 5. Interestingly, it is noted that among these three molecules, the structures of palbociclib and LEE011 are similar, and the major difference lies in the bicyclic core where palbociclib possesses a pyridopyrimidine and LEE011 has a pyrrolo-pyrimidine instead [84]. Actually, both of the two chemicals are highly specific inhibitors of CDK4/6, and are now under phase III clinical trials [85], in which a phase III study of palbociclib in combination with letrozole for women with ER(+)/HER2(−) advanced metastatic breast cancer is being carried out [85]. Phase III study of LEE011 in postmenopausal women with advanced breast cancer is being implemented [86]. During these studies, two questions that have been asked most frequently are, naturally, *Why do these inhibitors exhibit high selectivity to CDK4/6?* *And what are the implications for designing specific CDK2 inhibitors?* As a matter of fact, by structural comparison of the three kinases, it is observed that most residues in the ATP binding sites of CDK2, CDK4 and CDK6 are well conserved [83]. CDK4 and CDK6 resemble each other in some ways, while CDK2 always differs from them. A major difference is the presence of a histidine residue in His95 of CDK4 and His100 of CDK6 whose side-chains are in a specific position making both the kinase CDK4 and CDK6 easier to form a hydrogen bond with corresponding inhibitors, while in the equivalent position of CDK2, a phenylalanine residue (Phe81) takes the place of histidine [83]. When comparing CDK2 and CDK4, another difference is observed in their binding sites, that in CDK2’s binding pocket the three residues Lys89, His84 and Gln131 exist, whereas in CDK4’s pocket, in three corresponding equivalent positions, are residues Thr120, Asp97, Glu144, which all possess a negative charge relative to CDK2 [83]. In fact, relevant research has implied that charge may be responsible for the specificity of CDK4 inhibitor [87]. Additionally, structural analysis of CDK2 and CDK6 shows that small conformational differences in the hinge region of the two kinases are responsible for the inhibitor’s specificity by inducing changes in the ligand orientation, which results in sterical clashes in CDK2 but not in CDK6 [88]. If all these conformation differences between CDK2 and CDK4/6 are considered, it may hasten the process of developing specific CDK inhibitors.

**Figure 6:**
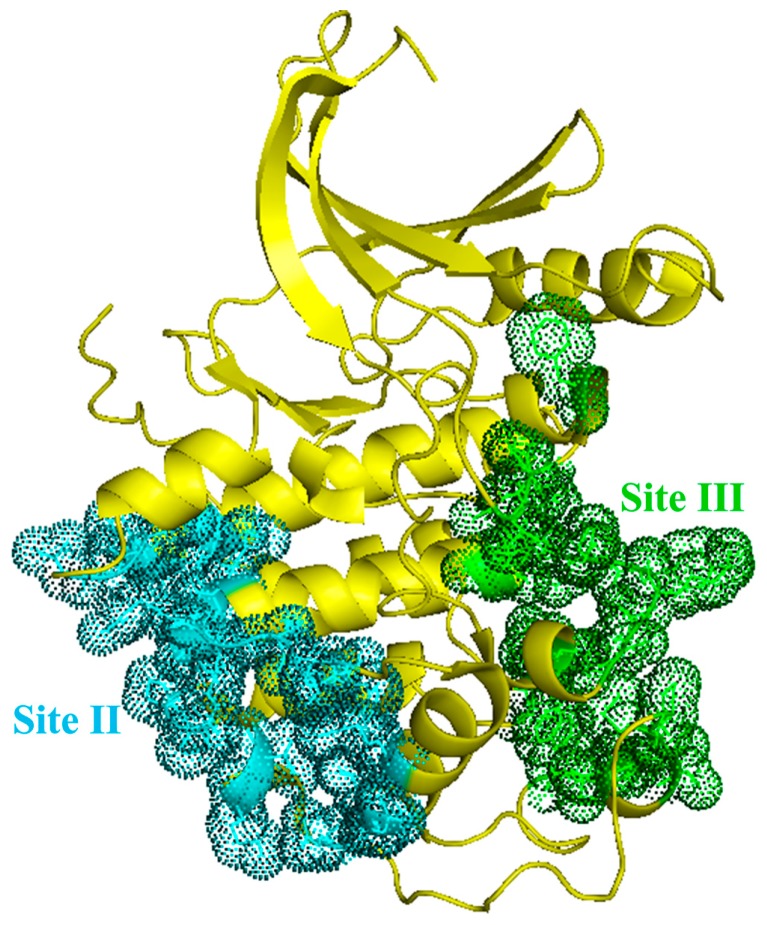
Non-competitive binding pockets of CDK2. Sites II and III are depicted in cyan and green, respectively. The amino acids in two pockets are provided in the text.

**II. Non-competitive binding sites (Site II andIII).** In the structure of CDK2, there also exist two non-competitive binding sites. These two sites are not in association with the catalytic subunits, and have been described in our previous work [80]. The second reported binding site “Site II” (as displayed in Figure 6) consists of amino acids at positions 97–101, 104, 194, 196–204, 214, 217–218, 246, 250–251, and 253–254. Thus far, however, our understanding is still quite limited and its binding affinity remains ambiguous [89]. The third discovered binding groove—“Site III” (as shown in Figure 6), which is composed of amino acids at positions 124, 152, 154–156, 172, 176–182, 184, 227–230, 232–234 and 270–272 [89], is proven to accommodate short peptides, such as TAALS, TAALD, TAALE, LAALS, TAACS and FAALS, that partially dissociate the CDK2/cyclin E complex, thus obstructing the enzymatic activity of CDK2 [89]. Experimental analysis demonstrates that short peptides which target this non-catalytic binding pocket can bind to both CDK2 and cyclin, and then interfere with the interface formation of the complex to pin down the substrate recruitment. Targeting the protein−protein interaction interface is a valid but also challenging strategy in that the interface typically spans a wide contact area without any obvious dominant contacts to target [90]. The top 10 residues involved in the formation of CDK2/cyclin interface in inactive states (when disassociated from its cyclin partner) rank as Tyr180, Glu208, Asp235, Lys178, Leu174, Arg126, Val154, Ile173, Gly176 and Arg150, while in active states (in complex with cyclin) rank as Tyr179, Trp227, Pro228, Pro155, Lys178, Tyr180, Val456, Pro271, Met233, Cys177 [89]. Among them, Lys178 and Tyr180 appear to be the favorite residues to target in Site III [89]. And there is evidence that peptides tend to bond to the active form of CDK2; however, it is not easy to block the formation of CDK2/cyclin E complex. Using the theoretical structure analysis of CDK1, CDK4 and CDK6, Site III at the interface is only detected in CDK1. Therefore, this suggests that the binding pocket at the interface, *i.e.*, Site III, may be less conserved and thus provides a more suitable and accessible target than the ATP binding site for designing relatively specific inhibitors of CDK2 [89].

### 2.2. Conformational Changes of CDK2 via Cyclin Binding

**Figure 7:**
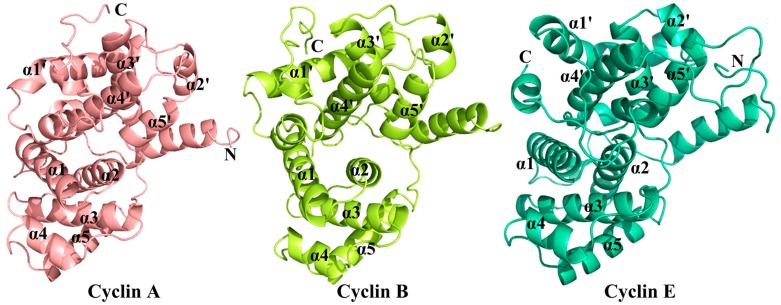
Schematic diagrams showing the structures of cyclins A, B and E, as well as their corresponding secondary structural elements. Coordinates used for cyclins A, B and E are 1OL1, 2JGZ and 1W98, respectively.

To date, three types of cyclins (A, B and E) have been crystallized successfully with CDK2. Cyclins A and E contain 260 (PDB code 1FIN, residues 173–432) [6] and 283 amino acids (PDB code 1W98, residues 96–378) [91], respectively. These cyclins activate their respective CDKs and confer diverse substrate recognition properties on CDKs [92]. For instance, studies prove that cyclin B confers M phase-like properties on CDK2 [92]. As can be seen from Figure 7, cyclin A comprises two five-helical cyclin boxes, *N*- and *C*-terminal domains. The structures of cyclins E1 and B [92] resemble that of cyclin A, both folding as two five-helical domains with additional *N*-terminal and *C*-terminal helices. The *N*-terminal helix of cyclin E (residues 126–225: α1–α5) even possesses identical structure and topology to that of cyclin A (residues 207–365). The center of the α3 helix is fully embraced by the other four helices and the whole part of the α3 helix comes into being as an organizing hydrophobic core of the motif. For the *C*-terminal cyclin box, significant conformation and sequence differences have been identified between cyclins A and E, especially in the length of the α1’ helix, despite these two cyclins having the superficial resemblance in the arrangement of the five α-helices [91]. Different from the disposition of the *N*-terminal cyclin box, the α3' and α4' of the *C*-terminal box are situated in the extended loop between α1' and α2' of cyclin E1, and the α5' is kinked at the *C*-terminal region.

**Figure 8:**
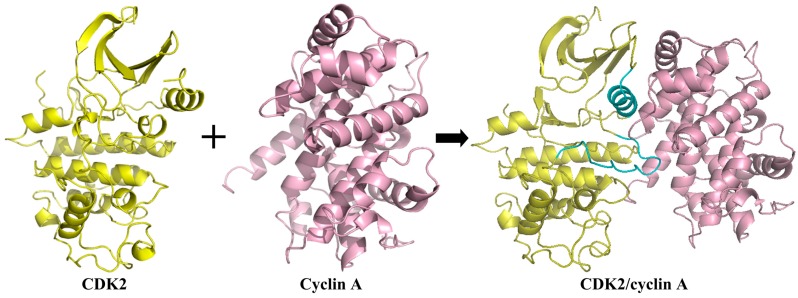
Formation of the CDK2/cyclin heterodimer. The cyan fragments are regions undergoing structural changes upon association with the cyclin subunit. Coordinates used for CDK2 and CDK2/cyclin A are 1HLC and 1OL1, respectively.

Cyclin binding at appropriate timing is one step for the activation of CDK2 [68], and the other step is the phosphorylation by the CDK-activating kinases [10,93], which will be explained in detail in next part. Cyclins contact with one side of the catalytic cleft and interact with both the N and C lobes of the kinase. The interaction of CDK2 and cyclin A is depicted in Figure 8. To accommodate cyclins well, CDK2 rearranges its structure, where the missing active site residues are restored to their correct positions. Additionally, most of the T-loop including both the structure and position is reconstructed, which contains rebuilding this flexible structure, and restoring missing active site residues to their correct positions. When cyclin binds to CDK2, the *N*-terminal domain, especially the αC-helix, shows some structural changes which lead to a slight broadening of the active site, and then releasing the catalytic cleft and exposing the phosphorylation site on the T-loop [92]. After structural reestablishment upon associations with cyclins, CDK2 is well-constrained mainly through hydrophobic interactions which are formed by residues of the cyclin linker between α5 and α1’ at the CDK2/cyclin interface with cyclins. However, the cyclin subunit holds essentially invariant conformation compared to the free unphosphorylated form [92]. The formation of dimer after elastic structural rebuilding generates a continuous protein−protein interface, and then facilitates further conformational changes in the region of the C-helix of the activation segment as well as in the relative orientation of the *N*- and *C*-terminal lobes, which finally result in a correct conformation for ATP triphosphate recognition site [6,66]. Integration with cyclins engenders great changes in the kinase structure. Taking cyclin A as an example, after it binds to CDK2 [6], the T-loop, which is the main obstacle of the substrate access in the monomeric apoenzyme, is displaced outside of the catalytic cleft exposing to the solvent. Moreover the PSTAIRE helix rotates by 90° and is moved into the catalytic cleft, thus allowing exposure of the activation segment in the region of the C-helix for Thr160 phosphorylation [1]. The crystal structures of CDK2/cyclin B and CDK2/cyclin E complexes with small ligands are not available for the present. There is evidence that in spite of the almost equivalent enzyme conformations extruded by cyclins B and A, the recruitment sites on the two cyclins and their functional features to promote the conformation activation are different [91,92]. The cyclin A recruitment site inclines to attract those substrates containing a RXL motif. Due to the sequence differences between cyclins A and B, binding interaction between cyclin B and RXL motif in this groove is relatively weak. And the canonical substrate recognition motif SPXK in cyclin A is found absent in cyclin B, which is replaced by the sequence SPXX in cyclin B. As a result, the CDK2/cyclin B phosphorylation is independent of the canonical sequence SPXK. As a blessing in disguise, both two sites on cyclin B could be phosphorylated while merely the Ser640 site can be phosphorylated on cyclin A. The reason is that the second site and the RXL motif are too close to cyclin A, which does not allow the cyclin A recruitment site to execute efficiently.

**III. Allosteric binding site (Site IV).** It is noteworthy that the conformational changes caused by the assembly of cyclin and CDK2 result in a novel binding site on CDK2, which is called as ANS (fluorophore 8-anilino-1-naphthalene sulfonate) binding pocket (*i.e.*, allosteric binding site, Site IV). This site is located in a region which is adjacent to the C-helix, away from the ATP site, and in fact located approximately halfway of the ATP site and the C-helix. The gatekeeper residues Phe80 and Lys33, along with the DFG motif, guard the ATP pocket to ensure that the ANS site extends above the helix, pointing away from the DFG region. Moreover, this site is independent from the ATP pocket. ANS quenches the interaction between CDK2 and cyclin, resulting in C-helix conformational fluctuations that are incompatible for cyclin A association [94]. ANS is the first known extrinsic compound which binds to this pocket with proved high affinity. Soon afterwards, some other agents were also screened out to hit this binding site [95]. Accommodation of these compounds makes the DFG region in CDK2 not subject to the structural variations derived from the integration with cyclin A, thereby resulting in the neighboring hydrophobic pocket still connected with the adjacent ATP site. However, when small compounds bind to the ATP site, Site IV becomes sensitive to substantial conformational transition. Similar to the mechanism of inhibition in Site III, molecules targeting at the ANS binding pocket dissociate cyclin A from CDK2, and are accompanied by significant changes of the activation loop and the DFG region. At the same time, they also make the enzyme incapable of interacting with cyclin A effectively, which finally inhibits the catalytic activity of the phosphorylated CDK2/cyclin A2 complex. Although it was proven that ANS specifically interacts with CDK2, it does not do so with cyclin A [94]. In this site, inhibitors are fixed by an elaborate network of H-bonds salt-bridge effects and hydrophobic interactions. It is necessary to point out that ANS molecules are susceptible to conformational fluctuations which are aroused by the taking of competitive inhibitors in the ATP binding pocket. However, due to the fact that the affinity of ANS for CDK2 (*K*_d_ = 37 μM) [94] is remarkably lower than that of cyclin A (*K*_d_ = 0.6 μM) [95], ANS is readily displaced from CDK2 upon the interaction with cyclin [96]. Therefore, in order to break the tight interaction of the CDK–cyclin interface, the allosteric inhibitors need to possess extraordinarily high inhibitory affinities [96].

### 2.3. Conformational Changes of CDK2 via Phosphorylation

**Figure 9:**
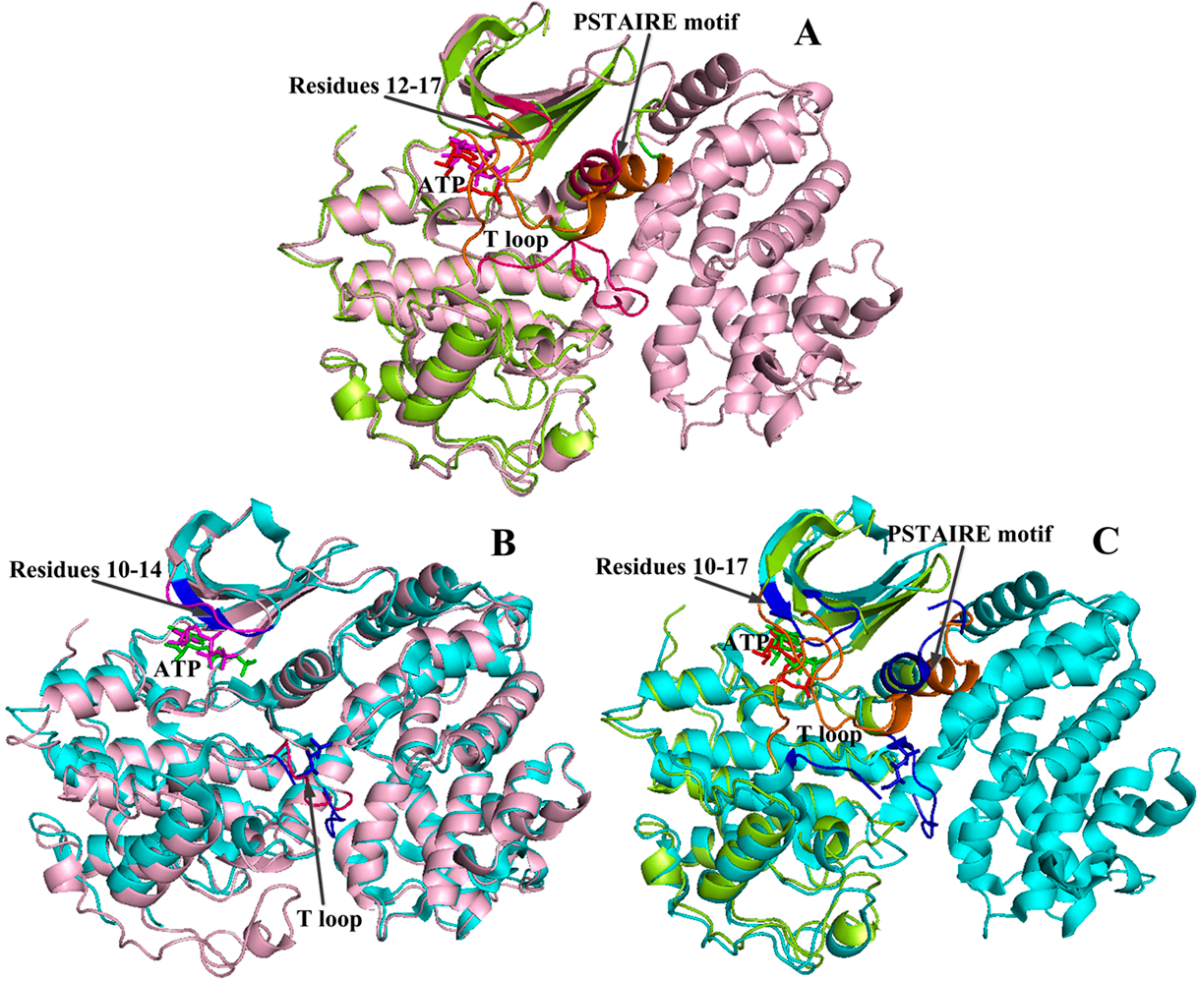
Comparison of the CDK2 conformation changes in free, partially and fully activated states. (**A**) Structural superposition of free and partially activated CDK2 complexes; (**B**) The alignment of partially and fully activated CDK2 complexes; (**C**) Structural superposition of free and fully activated CDK2 complexes. The yellow, pink and cyan patterns indicate the monomeric CDK2 (free state, coordinates 1HCK), CDK2/cyclin A complex (partially activated form, 1FIN) and phosphorylated dimer (fully activated state, 4EOM), respectively. The changed conformations of monomeric CDK2, CDK2/cyclin A complex and phosphorylated dimer are colored in orange, magenta and blue, respectively.

Phosphorylation on Thr160 is another factor that causes the conformational changes of CDK2, and it completes the reorganization of the substrate binding site that is initiated by the cyclin binding. This phosphorylation is carried out by a CDK-activating kinase (CAK) [97]. Cyclin binding to CDK2 converges the T-loop which includes the activating Thr160 into a more active-like conformation where the activating Thr160 becomes accessible for CDK phosphorylation [6]. The phosphorylation of Thr160 by CAK moves the T-loop further, enabling the phosphate group to interact with several amino acids, which stabilizes the T-loop structure [98]. Though cyclin binding is the important stage for the activation of CDK2, it has no effects on Thr160 phosphorylation of CDK2 [69]. However, phosphorylation of the CDK/cyclin dimer with the phosphorylated Thr160 which acts as a final organizing center makes the kinase in full activation state, and also makes the T-loop docked onto the C lobe [98,99]. In this step, the region centered within the activation segment is primarily subjected to a conformational disorder. Additionally, the phosphate group Thr160 turns into the organizing center of the T-loop, coming into contact with three arginine side chains (Arg50, Arg126 and Arg150), each of which forms interaction with a different part of CDK2 (one from the T-loop, the other two from the C and N lobes, respectively) [69]. In the unphosphorylated monomeric CDK2, Thr160 is located near Glu12 side chain, and the aromatic nucleus of Tyr159 stacks against the two-dimensional peptide backbone around Gly16. The structure of glycine loop is also enhanced by attached binding interactions between the backbones of residues Glu12-Thr14 and the ribose-triphosphate group of ATP [69]. However, by virtue of the steric and charge constraints, the phosphorylation of Thr160 impedes these stabilizing interactions between the amino acid residues in the activation segment and the glycine-rich loop [69]. And the mobility of those residues in the activation loop, including His161, Glu162, Val163, and Val164 is subsequently increased. Thus the changes that occur in the ATP binding pocket can also be observed, and the enzyme’s affinity for ATP has been raised. The significant differences between the fully active and monomeric CDK2 are the shift of the C-(PSTAIRE) helix and the correct location of the activation segment. However, the high hydrophobic character and the conformation of the CDK2 active site are not significantly modified by Thr160 phosphorylation. It is noteworthy that CDK2 can undergo further structural changes to accommodate endogenous or exogenous inhibitors, even when it is completely assembled and activated. To offer some intuitionistic information about the enzyme’s conformational variations among the free CDK2, the CDK/cyclin heterodimer and phosphorylated CDK2, Figure 9 which respectively show differences in free, partially activated and fully activated CDK2 are illustrated.

### 2.4. Comparison with Active CDK2/Cyclin Complexes

**Figure 10:**
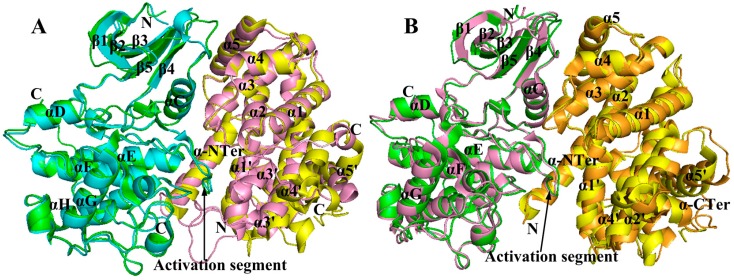
The structure of pCDK2/cyclin A superimposed on pCDK2/cyclin E1 and pCDK2/cyclin B, respectively. (**A**) Structural superposition of pCDK2/cyclin A (CDK2 in green and cyclin A in yellow, respectively) and pCDK2/cyclin E1 (CDK2 in cyan and cyclin E1 in pink, respectively); (**B**) The alignment of pCDK2/cyclin A and pCDK2/cyclin B (CDK2 in pink and cyclin B in orange, respectively). Coordinates used for CDK2/cyclin A, CDK2/cyclin B and CDK2/cyclin E1 are 1QMZ, 2JGZ and 1W98, respectively.

The CDK2/cyclin E complex is responsible for the G1-S transition through licensing the DNA origins of replication [100], and also regulates the centrosome duplication [74]. During the progression of the S phase, CDK2 associates with cyclin A, and this complex phosphorylates various substrates which allow the DNA replication and the inactivation of G1 transcription factors [74]. Cyclin B confers M phase-like properties to CDK2 [93], and CDK2/cyclin B can facilitate the G2/M progression [101]. Factually, the overall structures of the active pCDK2 (phospho-CDK2)/cyclin E1 and pCDK2/cyclin B are similar to the active pCDK2/cyclin A (as displayed in Figure 10) [91,92]. However, due to the different sequences of these cyclins, there are also some significant differences.

To compare the differences between pCDK2/cyclin A and pCDK2/cyclin E1, the structures of pCDK2/cyclin E1 superimposed on pCDK2/cyclin A are displayed in Figure 10A. As in pCDK2/cyclin A, the C-helix PSTAIRE packing on one side is almost parallel to the cyclin E1 α5 helix and is nearly vertical to the *C*-terminal end of the cyclin E1 α3 helix [91]. In pCDK2/cyclin E1, the principle contact residues include those from the *N*-terminal helix, helices α3, α4, α5 and α1', the loop between α5 and α1', the loop from α1' and α2', as well as that from the *C*-terminal region. There are extensive hydrophobic interactions, especially those ones with pCDK2 Ile49 and Ile52. Moreover, the *C*-terminal residues wrap around the outside of the kinase and come close to the αD helix (residues 87–95) where Leu296 stacks against Phe90. In the structure of pCDK2/cyclin E1, the direction of the polypeptide chain from residue 289 onwards is almost 180° as compared with that in the pCDK2/cyclin A complex, and residues 289–297 make no contact with pCDK2 structure [91]. In its rotated conformation, the PSTAIRE is conducive to the stabilization of the triphosphate moiety of ATP in the correct position for catalysis by Lys33 with a hydrogen bond connecting with Glu51. As a result of the sequence changes, the 40s loop (residues 33–41) that immediately precedes the PSTAIRE helix makes contacts with cyclin E1 in a different way from that of pCDK2/cyclin A [91], the conformational variation of pCDK2 loop and hydrogen bonding partners at the interface (cyclin E1 residues Gly208 and Asp209 displace the Lys288 and Lys289 of cyclin A) bring about degradation of close contacts with pCDK2 (cyclin E Asp209 and pCDK2 Glu40). Moreover, in the shifted position of the 40s loop, Leu216 of cyclin E1 can contact Leu37 in pCDK2 [91]. In addition, by comparison with pCDK2/cyclin A, the pCDK2 70s loop region (β4/β5 loop; residues 71–74) also shifts with a change of His296 in cyclin A to Leu216 in cyclin E1, making it suffer the loss of interaction between the leucine with the pCDK2 His71 and Thr72 [91]. As for the protein−protein interface, the pCDK2/cyclin E1 surface is further strengthened when being compared to the pCDK2/cyclin A by additional interactions in the vicinity of the *C*-terminal cyclin box, including hydrophobic interactions especially with pCDK2 Ile49 and Ile52. Despite some sequences changing, the interface is glued by hydrogen bonds in a similar pattern to the pCDK2/cyclin A complex, and the pCDK2 40s and 70s loop regions are situated in alternative conformations to cover the sequence changes without affecting the pCDK2 catalytic residues. In pCDK2/cyclin A complex, the side chain of pCDK2 Lys56 contacts with cyclin E1 Arg305, but in cyclin E1, the corresponding residue is Arg225, and both basic groups of the pCDK2/cyclin E1 dimer (pCDK2 Lys56 and cyclin E1 Arg225) are exposed and kept away from one another [91]. The additional contacts made by α1’/α2’ loop and the *C*-terminal region contribute to increasing the buried molecular surface area in pCDK2/cyclin E1 when being compared to pCDK2/cyclin A.

Cyclin B1 is also proved to be able to form a stable complex with pCDK2, and generate an active structure, which is almost identical to that of pCDK2/cyclin A in the structural and biochemical studies [92]. For comparison, structural superposition of pCDK2/cyclin A and pCDK2/cyclin B is shown in Figure 10B. As in pCDK2/cyclin B, the residual conformation of the catalytic site which gets involved in the ATP recognition, catalysis, and the activation segment for protein substrate recognition, is similar with that in pCDK2/cyclin A [92]. Moreover, there are even no significant sequence and structural changes in the surface of the cyclins that adjoin the peptide substrate recognition site and the side chain conformations in the vicinity [92]. The exceptions are 40s, 70s and the glycine rich loops that arise from the sequential difference between cyclins B and A causing different interactions. Yet, after all, the array of residues in cyclins B and A is very different, the pCDK2/cyclin B shows its own characteristics. The loops in the pCDK2/cyclin B dimer bind a little bit closer to the catalytic site of pCDK2, spontaneously followed by the conformational differences in kinases. However, it is noteworthy that the residues contained in these three external loops are among the least well ordered in pCDK2 and their conformation is significantly less conserved. This is not only due to the cyclin sequences, but also to the different environments or ligand complexes in the crystal lattice. The pCDK2/cyclin B exhibits substrate specificity. There exist differences between cyclins A and B at the recruitment site, which in cyclin A is accustomed to recruit substrates that contain an RXL motif. Due to the sequence differences, this site of cyclin B targets RXL motif more weakly than that in cyclin A [92].

The pCDK2 activation segment (residues 145–171) is the key element of protein substrate recognition. The cyclin in concert with phosphorylation has a major role in the correct alignment of this activation segment [92]. As in the pCDK2/cyclin E1 complex, the phosphor-Thr160 contacts three arginine residues from CDK2 (Arg50, Arg126 and Arg150), and acts as an organizing center to locate this activation fragment in concert orientations [86]. And these interactions are further enhanced by two hydrogen bonds formed with cyclin E1 Leu187 and Glu188, whose main chain carbonyl oxygens contact with CDK2 Arg50 and Arg150, respectively [92]. This arrangement is similar to that in pCDK2/cyclin A dimer [91]. Further comparison of the structural differences among pCDK2/cyclin A, pCDK2/cyclin B and pCDK2/cyclin E1 may be helpful to understand their differential biological roles in governing the cell cycle progression.

### 2.5. Changes of the Interaction via Conformational Fluctuations

**Figure 11:**
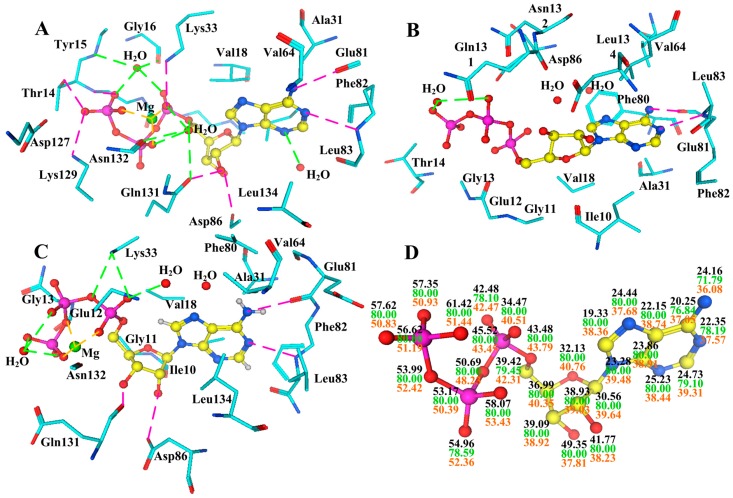
Variations of the ATP binding site through conformational fluctuations. (**A**) is the interaction network of ATP in monomeric CDK2 (coordinates 1HCK, resolution 1.9 Å); (**B**) is in CDK2/cyclin A dimer (1FIN, 2.3 Å); (**C**) is in the phosphorylated dimer (4EOM, 2.3 Å), respectively; (**D**) The B-factor (Å^2^) distribution for the ATP atoms across the monomeric CDK2 (in black font) CDK2/cyclin A complex (in green) and phosphorylated dimer (in red).

Due to the structural arrangement of the two key elements (the C-helix and the activation segment) in the inactive monomeric CDK2, residues in the ATP binding site are erroneously disposed, which cannot support the correct alignment of the triphosphate moiety for catalysis, though this monomer can bind to ATP [66]. In the free CDK2, as shown in Figure 11A, the adenine group is fixed by three H-bonds, *i.e.*, the ones with Glu81 carbonyl, Leu83 amine group and one water molecule, respectively. Apparently, two conserved H-bonds are well reserved in this subsite. And the restraints of the ribose moiety are also two H-bonds, with the two hydroxide radicals separately connecting to the Gln131 carbonyl and Asp86 carboxyl groups. The triphosphate group of ATP is mainly confined by four H-bonds in a fan-shaped pattern, and one salt-bridge interaction via the Mg^2+^ cation. The four H-bonds are formed with the NH^3+^ cation of residues Lys33 and Lys129, hydroxyl and amide groups of Thr14. Even more, two water molecules appear in the cavity and are involved in the interaction network to generate solvent contacts.

Owing to the particular situation of the ATP binding pocket at the domain–domain interface, the conformational changes of CDK2 followed by the exogenous variables inevitably lead to the variation of this pocket. Upon cyclin binding, large conformational changes of CDK T-loop expose the catalytic sites of CDK in preparation for the touchdown of potential substrates [6] and unmask the triphosphate recognition site for further activation [1,6], followed by some variations occurring in the ATP binding site. The adenine subpocket of CDK2 is conserved. However, the ribose subpocket may be subjected to significant perturbation as a consequence of the transformation of the glycine loop upon cyclin binding. Besides, the interaction network including H-bonds and electrostatic interactions in the triphosphate region also goes through some subtle variations. In detail, the amino acid side chains within the ATP binding site are reoriented. For example, Glu51 instead of Lys33 is directed into ATP binding site. What is more, the less conserved residues His84 and Gln85 outside the ATP binding pocket also undergo significant movements upon the cyclin binding. The interaction of CDK2 and substrate also changes, which is evidenced by the interaction of CDK2 and ATP as shown in Figure 11B. At this time, the whole acting force is impaired, and the two H-bonds in the ribose region and four in triphosphate region fade away. In addition, the Mg^2+^ cation disappears in the cleft, and thus the relevant metal contacts are degraded, despite the fact that two conserved H-bonds still persist in the adenine region. Besides, only one water molecule that shows up in the triphosphate subsite generates a solvent contact in supplement of the two H-bonds in the adenine region.

As for the fully activated CDK2 in Figure 11C, the interaction mechanism is similar to that of the free CDK2. The two conserved H-bonds in the adenine subsite connecting with Glu81 carbonyl and Leu83 amine groups remain in this region. Additionally, the two H-bonds in the ribose subpocket are almost identical to those in the monomeric CDK2’s ATP site. Besides, the ionic interaction in correlation with Mg^2+^ cation and the water-related solvent contacts also turn up in this binding fashion. However, the H-bond effects are attenuated in the triphosphate region, with only two H-bonds generated by the NH^3+^ moiety of Lys33 observed, one of which is the same as the one appearing in the monomeric CDK2’s triphosphate region.

Owing to the structural arrangement of the C-helix and the activation segment in the inactive CDK2 monomer, residues in the ATP binding site are erroneously disposed. The Thr160 among the activation segment conformation is buried away from solvent. After cyclin A binds to CDK2, large conformational changes of the activation segment relieve the catalytic cleft and expose the phosphorylation site in preparation for the touchdown of potential substrates. Nevertheless, though cyclin binding is an important step for CDK2 activation, it has no effects on Thr160 phosphorylation of CDK2. Subsequently, phosphorylation of the CDK/cyclin complex with the phosphorylated Thr160 makes the activation segment dock onto the C lobe [98,99]. Subsequently the Thr160 phosphate group turns into the organizing center of the activation segment, which then has interactions with three arginine side chains. For further research, the B-factor distributions for the ATP atoms across the free, partially and fully activated structures are displayed in Figure 11D. The comparative result displays that the phosphorylated CDK2/cyclin A complex with low B-factor distribution of ATP atoms has a more stable structure.

**Figure 12:**
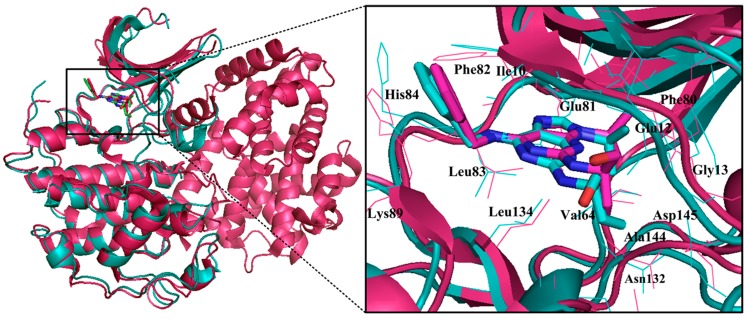
Comparison of the structures of the free and phosphorylated CDK2 dimer in complex with roscovitine. The cyan and fuchsia patterns indicate the monomeric CDK2 (coordinates 2A4L) and the phosphorylated CDK2/cyclin A dimer (3DDQ) in a complex with roscovitine, respectively.

**Figure 13:**
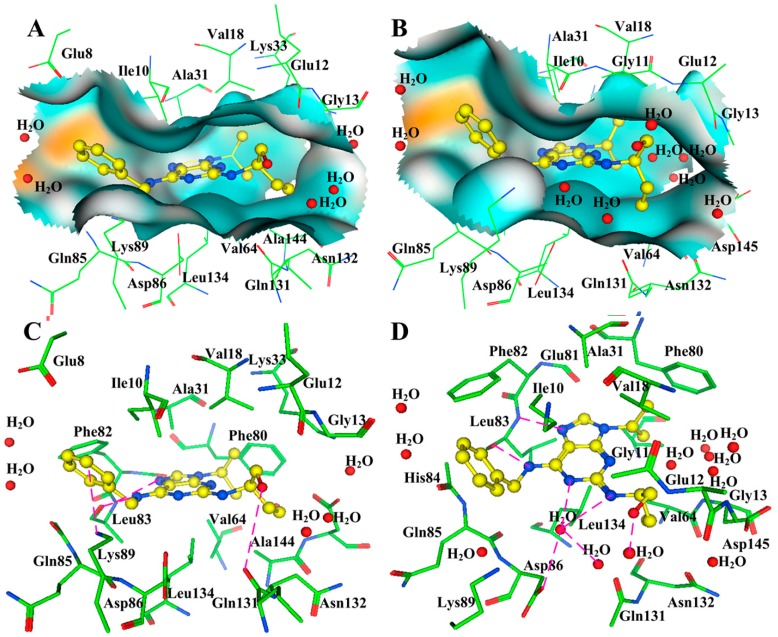
The binding pockets and interaction mechanisms of roscovitine in free and phosphorylated CDK2 dimers. (**A**,**C**) are for the monomeric CDK2 (coordinates 2A4L); (**B**,**D**) are for the phosphorylated CDK2 state (3DDQ), respectively.

In fact, the structural fluctuations via cyclin binding and phosphorylation also bring about some perturbation for the interaction of CDK2 with small ligands. The rough information about the conformational difference of roscovitine in a complex with free and fully activated CDK2 is provided by the alignment of the two crystals in Figure 12. It can be observed that the small molecule makes some small conformational transformation fit into the novel binding pocket after phosphorylation of the apoenzyme. The information provided in Figure 13 offers more details about the turbulence in the interaction mechanism of the two states of CDK2. From the perspective of the appearance, the shape of the semi-open pocket goes through alterations. When it comes to details, the specific interactions also vary according to the movements of both the residues and the ligand. In the catalytic site of the monomeric CDK2, the purine core is stabilized by two H-bonds formed with the carbonyl and amino groups of Leu83. The phenyl group on N_6_ is constrained by an arene−cation interaction with the NH_3_^+^ cation of Lys89, and another substituent on N_2_ is restricted by a water molecule with a direct H-bond. van der Waals contacts play a subsidiary role in fixing the numerous atoms of the ligand. As for the situation in the phosphorylated dimer, two conserved H-bonds formed by the Leu83 are reserved despite the small changes in distances. However, the arene−cation interaction is resolved, and the H-bond formed with the N_2_ substituent is also degraded. Instead, some solvent contacts arise near N_1_ and N_6_ positions of the purine core.

## 3. Conclusions

Being one member of the highly conserved protein kinases, CDK2 is a pivotal regulator of the eukaryotic cell cycle. However, its monomer is inactive in the quiescent cells, which needs to be aroused by specific partners containing cyclins A and E, or be phosphorylated on the catalytic region. Significantly, these activated processes can lead to conformational changes in and around the catalytic domain of CDK2, which reflect the intrinsic conformational flexibility of CDK2. This flexible nature is crucial for switching the states of CDK2 in responses to different regulatory signals which are implicated in eukaryotic cell cycle. Therefore, studies on the structural characteristics as well as the ligand binding mechanisms of CDK2 will enhance our understanding of the molecular mechanisms regulating the activities of this kinase, and may also provide useful implications for developing specific and powerful CDK2 inhibitors.

In the CDK2 monomer, three binding sites have thus far been reported, including the ATP-competitive binding site (Site I) and two non-competitive binding sites (Site II & III). Among them, Site I, the prime target site of CDK2 inhibitors, is conserved and has the impressive capacity to accommodate various ligands containing flat heterocyclic rings. As for Site II, its binding mechanism remains ambiguous, because of the deficiency of existing relevant research. Fortunately, Site III that accommodates short peptide ligands may be less conserved and thus provides a more suitable target to design specific CDK2 inhibitors. Additionally, when CDK2 is subjected to the cyclin binding process, the resulted conformational changes give rise to a variation of the ATP binding site, and then generate an allosteric binding site, *i.e.*, Site IV, which requires the binding inhibitors to possess extraordinarily high inhibitory affinities. In summary, for designing more effective CDK2 inhibitors, we can adopt the following principles: (1) developing inhibitors with flat heterocyclic rings for ATP binding site; (2) designing short peptide ligands for Site III; and (3) employing binding molecules which have extraordinarily high inhibitory affinities for Site IV. Nevertheless, the high sequence homology within the ATP binding site of CDKs makes the ATP-competitive inhibitors exhibit low specificity. Therefore, further research on binding mechanisms on Site III and IV may provide more information to develop highly selective inhibitors for CDK2.
